# Dose-dense and less dose-intense Total Therapy 5 for gene expression profiling-defined high-risk multiple myeloma

**DOI:** 10.1038/bcj.2016.64

**Published:** 2016-07-29

**Authors:** Y Jethava, A Mitchell, M Zangari, S Waheed, C Schinke, S Thanendrarajan, J Sawyer, D Alapat, E Tian, C Stein, R Khan, C J Heuck, N Petty, D Avery, D Steward, R Smith, C Bailey, J Epstein, S Yaccoby, A Hoering, J Crowley, G Morgan, B Barlogie, F van Rhee

**Affiliations:** 1 Myeloma Institute, University of Arkansas for Medical Sciences, Little Rock, AR, USA; 2 Cancer Research and Biostatistics, Seattle, WA, USA

## Abstract

Multiple myeloma (MM) is a heterogeneous disease with high-risk patients progressing rapidly despite treatment. Various definitions of high-risk MM are used and we reported that gene expression profile (GEP)-defined high risk was a major predictor of relapse. In spite of our best efforts, the majority of GEP70 high-risk patients relapse and we have noted higher relapse rates during drug-free intervals. This prompted us to explore the concept of less intense drug dosing with shorter intervals between courses with the aim of preventing inter-course relapse. Here we report the outcome of the Total Therapy 5 trial, where this concept was tested. This regimen effectively reduced early mortality and relapse but failed to improve progression-free survival and overall survival due to relapse early during maintenance.

## Introduction

Definitions of high-risk multiple myeloma (HiRMM) vary widely and include plasma cell leukemia, high lactate dehydrogenase, high tumor burden (ISS 3) and a number of genetic features.^1, 2, 3, 4, 5^ The latter include metaphase cytogenetic abnormalities, interphase fluorescence *in situ* hybridization abnormalities such as del17p, amp1q, t(4;14) and t(14;16) and t(14;20), as well as gene expression profiling (GEP)-based signatures and mutational pattern.^6, 7, 8^ We have previously reported that GEP70-defined high risk was the dominant adverse parameter with the highest early relapse rates, indicating that GEP70 accounted for most of the observed variability in clinical outcomes.^9^ In successive Total Therapy (TT) trials, we have noted a progressive improvement in clinical outcomes with the addition of thalidomide in Total Therapy 2 (TT2), incorporation of both bortezomib and thalidomide in Total Therapy 3a (TT3a) and lenalidomide and bortezomib as maintenance in Total Therapy 3b (TT3b).^10, 11, 12^ Unfortunately, this progress was limited to the 85% of patients with GEP70-defined low-risk MM.^13^ Lack of progress in the treatment of HiRMM prompted us to explore less intense dosing allowing greater dose density, and thereby minimizing drug-free intervals during which relapses had been noted in prior TT protocols. We report the outcomes of GEP70 high-risk subjects enrolled to Total Therapy 5 (TT5) and its comparison with outcomes of GEP70 high-risk in TT3a and TT3b patients.

## Patients and methods

Eligible patients were either untreated or had no more than one cycle of prior therapy for symptomatic MM fulfilling CRAB criteria. Subjects up to the age of 75 years were eligible, provided they had adequate cardio-pulmonary functions; liver function tests could not exceed twice normal values. Treatment assignment was done once GEP results were available.

**Figure 1:**
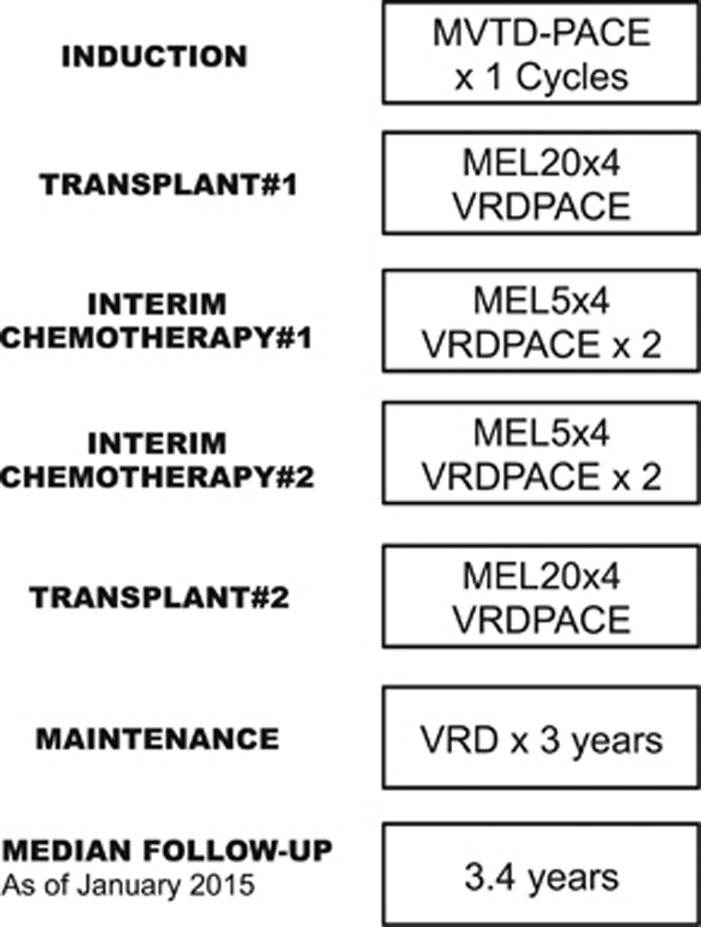
Total Therapy 5 treatment schema.

In addition to commonly employed MM marker analysis and bone marrow morphological examinations, we documented GEP70-based risk scores and molecular subgroups as well as fluorescence *in situ* hybridization-based del17p.^12^ Seventy-four patients were screened, of whom 50 were eligible for the enrollment into the study; 24 patients treated on the protocol were excluded from the final analysis because they were classified as low risk by GEP70. The treatment consisted of eight-drug combinations for induction (M-VTD-PACE; melphalan, bortezomib, thalidomide, dexamethasone; and four-day continuous infusions of cisplatin, doxorubicin, cyclophosphamide, etoposide), both transplants (MEL80-VRD-PACE (R, lenalidomide)), and two inter-transplant cycles with Mel20-VTD-PACE; followed by 3 years of maintenance with VRD (V=bortezomib, R=lenalidomide, D= dexamethasone) alternating with VMD (M=melphalan) (Figure 1). Owing to cumulative hematological toxicity from the VMD component seen with the first patients initiating the maintenance phase, VRD was later employed as the sole maintenance, with provision for bortezomib escalation to 1.5 mg/m^2^ weekly and of lenalidomide to 25 mg for 21 days of a 28-day cycle. The protocol and its revisions had been approved by the institutional review board. All patients signed a written informed consent acknowledging the investigational nature of the protocol, in keeping with institutional, federal and Helsinki Declaration guidelines. A Data Safety and Monitoring Board conducted annual reviews. A team of experts provided semiannual audits for protocol adherence, toxicities and efficacy data. Toxicities were graded according to Version 3 of the NCI Common Terminology Criteria for Adverse Events.

## Results

**Figure 2:**
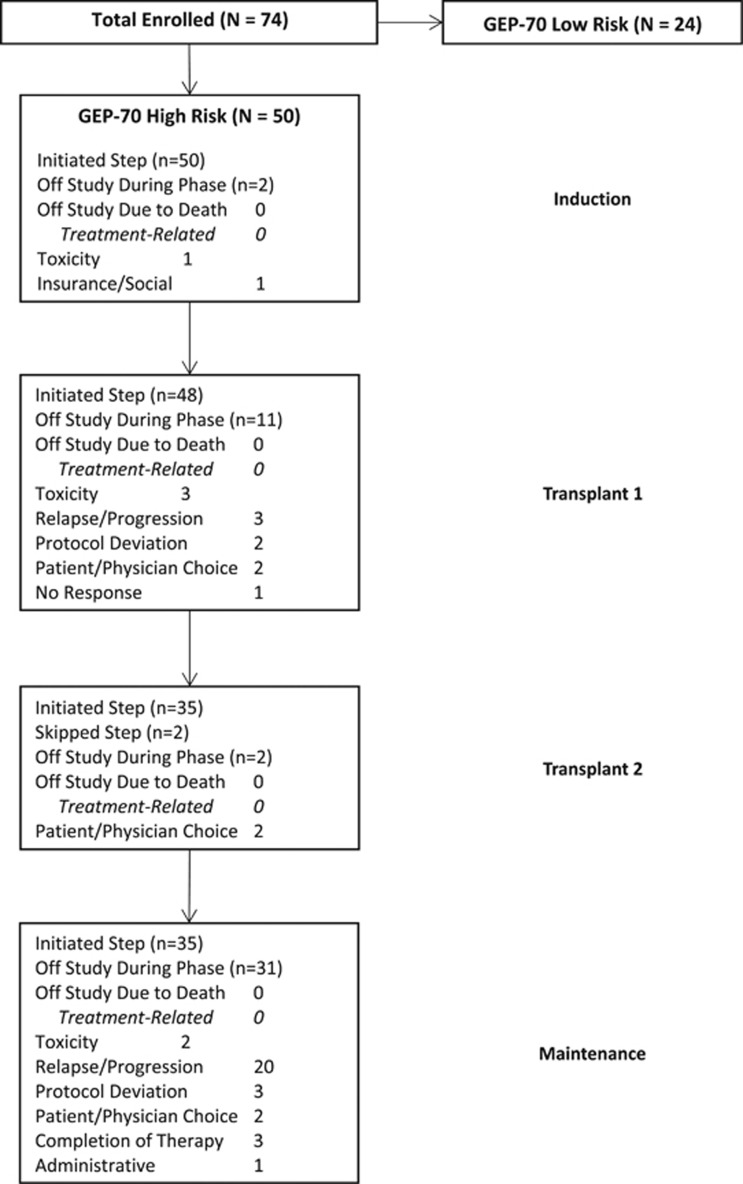
Consort diagram of patients enrolled in TT5.

**Figure 3:**
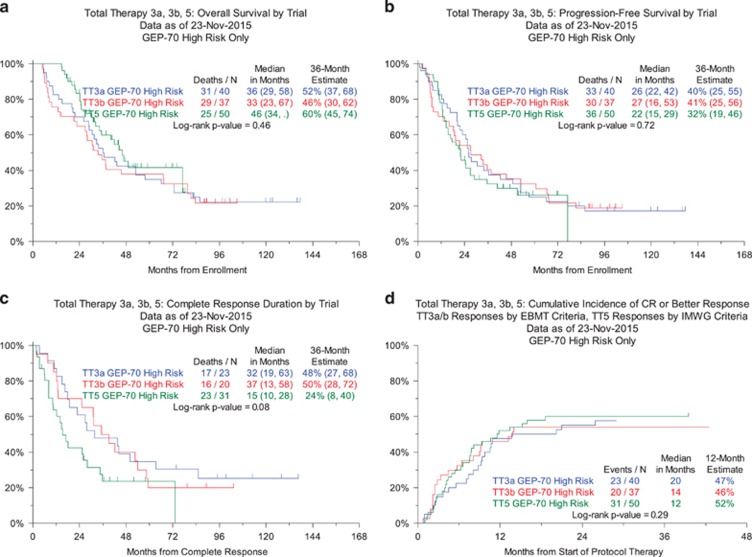
Outcomes for patients entered into TT5. (**a**) OS, (**b**) PFS, (**c**) CRD, (**d**) Cumulative incidence of response.

We report TT5 outcomes in the context of predecessor trials TT3a and TT3b for HiRMM, which both employed two inductions with VDTPACE, tandem transplantation with melphalan 200 mg/m^2^ and two dose reduced VDTPACE consolidations. The baseline characteristics of GEP70 high-risk patients in TT3a, TT3b and TT5 were comparable in terms of age, ISS stage, presence of PET-defined focal lesions (PET-FL, except difference in proportions with PET-FL-SUVmax>3.9) and presence of cytogenetic abnormalities (Table 1). The progression through sequential TT phases was, as intended, faster in TT5 than in TT3a and TT3b. A consort diagram depicts the progression of GEP70 high-risk patients through the individual trial components and lists reasons for drop-out (Figure 2). With median follow-up times of 10 years in TT3a, 7.6 years in TT3b and 4.4 years in TT5, 3-year estimates of overall survival (OS) were similar at 52, 46 and 60%, respectively (Figure 3a). The corresponding progression-free survival estimates (PFS) were 40, 41 and 32% (Figure 3b). The 3-year estimated complete response durations were virtually identical in TT3a and TT3b at 48 and 50% but tended to be inferior at 24% in TT5 (log-rank *P*=0.08) (Figure 3c). The timing of onset of complete remission was virtually identical among the three trials (Figure 3). We examined the association of baseline parameters with OS and PFS in TT5 through Cox regression analysis (Supplementary Table S2).

In univariate models, low albumin and B2M⩾3.5 mg/l both were associated with significantly shorter OS and PFS, while the 16% of patients with ISS I fared significantly better. Lower hemoglobin levels <10 g/dl were associated with shorter OS and PFS, while high CRP⩾8 mg/l was linked to shorter PFS. Clinical outcomes were almost identical across all GEP molecular subclasses. On multivariate analysis, hypo-albuminemia <3.5 g/dl remained the sole significant variable affecting both OS and PFS adversely.

The design of TT5 compared with TT3 trials emphasized dose density at the cost of dose intensity so that treatment-free intervals were minimized, which was accomplished (Figure 1). While pre-maintenance disease escape was rare in TT5, relapse occurred more frequently and promptly during maintenance than observed in TT3a and TT3b, consistent with a lesser depth of response from the pre-maintenance therapies used in TT5. Grade 3–5 toxicities and adverse events are summarized in Supplementary Table S1. Hematological toxicities such as thrombocytopenia, neutropenia and low hemoglobin were the commonest grade 3–5 toxicities observed in approximately 50% of the patients. There were no major infectious complications and, overall, the treatment was tolerated well.

## Discussion

TT5 was designed to allow for rapid sequential application of chemotherapy and transplants to sustain tumor kill by avoiding treatment-free periods as required after standard melphalan dosing at 200 mg/m^2^. While the intended timely sequencing of TT5 treatment segments was accomplished at acceptable toxicities (off study rate for toxicities, 12%), clinical outcomes were similar in TT5 and predecessor trials TT3a and TT3b, with a strong trend for inferiority for complete response durations despite comparable CR kinetics. This is consistent with what would be expected if TT5 produced a lesser depth of CR and may also explain why 20 of 35 patients experienced rapid disease progression during maintenance.

Owing to the dose-dense approach in TT5, patients received less cumulative chemotherapy than their TT3 counterparts. It is reasonable to speculate that conditioning with MEL80VDRPACE was less cytoreductive than melphalan 200 mg/m^2^, so that the well-documented melphalan dose–response was not executed. A further contributing factor to TT5's poor performance may be the omission of consolidation therapy. We had previously reported the importance of post-transplant consolidation even prior to the era of novel drugs.^14^ However, the similar PFS outcomes in TT3 and TT5 suggest that post-transplant consolidation in HiRMM merely postpones relapse, but does not prevent it. It is possible that the repeated application of suboptimal doses of melphalan in TT5 may have fostered genomic instability, but we do not have data to support this speculation. Both the TT3 and TT5 data suggest that HiRMM can initially be effectively treated but likely allows for the emergence of drug-resistant sub-clones. It is possible that the repeated application of suboptimal doses melphalan in TT5 may have fostered genomic instability, but we do not have data to support this speculation.

Taken together these data show, for stringently defined GEP70 HiRMM, that a dose-dense approach including tandem transplantation is not associated with long-term disease-free outcome and cure is only likely seen in 15–20% of patients who developed stable plateaus of response from 5 years. This is an unacceptable outcome that clearly needs to be improved. Promising options include monoclonal antibodies targeting myeloma directly or, alternatively, activating immune cells such as activated natural killer cells and CAR-T cells.^15^ Additionally, some patients may benefit from adding targeted therapy directed at N-RAS, K-RAS or BRAFF according to their mutational profile.^16^

## Figures and Tables

**Table 1:** Patient characteristics at presentation

*Factor*	*Analysis population*			P*-value*
	*TT3a GEP-70 High Risk*	*TT3b GEP-70 High Risk*	*TT5 GEP-70 High Risk*	
Median age (years)	56.3 (*N*=40) (36.2–74.7)	60.5 (*N*=37) (36.3–71.2)	61.3 (*N*=50) (38.1–74.0)	0.297^a^
Age>65 years	9/40 (23%)	12/37 (32%)	16/50 (32%)	0.526
Female	17/40 (43%)	20/37 (54%)	20/50 (40%)	0.401
White	35/40 (88%)	34/37 (92%)	44/50 (88%)	b
ISS Stage 1	11/40 (28%)	4/37 (11%)	8/50 (16%)	0.150
ISS Stage 2	9/40 (23%)	12/37 (32%)	17/50 (34%)	0.448
ISS Stage 3	20/40 (50%)	21/37 (57%)	25/50 (50%)	0.786
Creatinine⩾1.5 mg/dl	8/40 (20%)	8/37 (22%)	13/50 (26%)	0.781
Hemoglobin<10 g/dl	19/40 (48%)	22/37 (59%)	33/50 (66%)	0.206
LDH⩾190 U/l	21/40 (53%)	16/37 (43%)	19/50 (38%)	0.385
Platelet count<150 × 10^9^/l	8/40 (20%)	16/37 (43%)	22/50 (44%)	0.029
Baseline PET-FL>0	32/40 (80%)	26/37 (70%)	31/47 (66%)	0.328
Baseline PET-FL>3	23/40 (58%)	21/37 (57%)	24/47 (51%)	0.803
Baseline FL-SUV>3.9	26/32 (81%)	11/26 (42%)	18/31 (58%)	0.007
Baseline EMD	3/40 (8%)	3/37 (8%)	6/50 (12%)	b
Cytogenetic abnormalities	28/40 (70%)	28/36 (78%)	37/50 (74%)	0.742
GEP70 High Risk	40/40 (100%)	37/37 (100%)	50/50 (100%)	
FISH del17p	4/17 (24%)	9/30 (30%)	9/46 (20%)	0.583
FISH amp1q21	14/20 (70%)	16/30 (53%)	34/46 (74%)	0.172

Abbreviations: FISH, fluorescence *in situ* hybridization; LDH, lactate dehydrogenase; *n*/*N* (%): *n*—number with factor, *N*—number with valid data for factor; ND, no valid observations for factor.

a *P*-value from Kruskal–Wallis test.

b Sample size assumption for the *χ*^2^ test is not met.
