# Supplementary material for: Dose-dense and less dose-intense Total Therapy 5 for gene expression profiling-defined high-risk multiple myeloma
Source: Blood Cancer J. 2016 Jul 29;6(7):e453–. doi: 10.1038/bcj.2016.64 (PMC5030385; doi:10.1038/bcj.2016.64)
Supplement: Supplementary Table 1 [file bcj201664x1.doc]

**Supplementary Table 1.** Grade 3-5 Adverse Events by System Organ Class

| *System Organ Class* | *TT5 - GEP70 High Risk (N=50)* |
| --- | --- |
| AUDITORY / EAR | 5 (10%) |
| BLOOD/BONE MARROW | 50 (100%) |
| CARDIAC ARRHYTHMIA | 7 (14%) |
| CARDIAC GENERAL | 13 (26%) |
| CONSTITUTIONAL SYMPTOMS | 7 (14%) |
| DERMATOLOGY / SKIN | 3 (6%) |
| ENDROCRINE | 1 (2%) |
| GASTROINTESTINAL | 12 (24%) |
| HEMORRHAGE / BLEEDING | 1 (2%) |
| HEPATOBILIARY / PANCREAS | 14 (28%) |
| INFECTION | 23 (46%) |
| INFECTION, CLINICALLY DOCUMENTED | 1 (2%) |
| INFECTION, NORMAL ANC | 1 (2%) |
| METABOLIC/LABORATORY | 48 (96%) |
| MUSCULOSKELETAL / SOFT TISSUE | 15 (30%) |
| NEUROLOGY | 10 (20%) |
| OCULAR / VISION | 2 (4%) |
| PAIN | 17 (34%) |
| PULMONARY / UPPER RESPIRATORY | 5 (10%) |
| RENAL / GENITOURINARY | 1 (2%) |
| SECONDARY MALIGNANCY | 3 (6%) |
| VASCULAR | 2 (4%) |
