# Supplementary material for: Dose-dense and less dose-intense Total Therapy 5 for gene expression profiling-defined high-risk multiple myeloma
Source: Blood Cancer J. 2016 Jul 29;6(7):e453–. doi: 10.1038/bcj.2016.64 (PMC5030385; doi:10.1038/bcj.2016.64)
Supplement: Supplementary Table 2 [file bcj201664x2.doc]

**Supplementary Table 2.**

|  | | | **OS** | | **PFS** | |
| --- | --- | --- | --- | --- | --- | --- |
|  | **Variable** | **n/N (%)** | **HR (95% CI)** | **P-value** | **HR (95% CI)** | **P-value** |
| Univariate | Age >= 60 Years | 28/50 (56%) | 0.89 (0.40, 1.95) | 0.761 | 1.14 (0.59, 2.24) | 0.693 |
|  | Age > 65 Years | 16/50 (32%) | 1.50 (0.64, 3.51) | 0.351 | 1.41 (0.70, 2.84) | 0.337 |
|  | Female | 20/50 (40%) | 0.89 (0.39, 2.03) | 0.787 | 0.61 (0.30, 1.26) | 0.176 |
|  | White | 44/50 (88%) | 1.21 (0.36, 4.08) | 0.752 | 0.93 (0.36, 2.41) | 0.887 |
|  | IgA | 15/50 (30%) | 0.72 (0.30, 1.74) | 0.460 | 0.91 (0.43, 1.89) | 0.794 |
|  | IgG | 26/50 (52%) | 1.49 (0.68, 3.30) | 0.319 | 1.03 (0.53, 2.00) | 0.932 |
|  | ISS Stage 1 | 8/50 (16%) | 0.00 (0.00, .) | 0.004 | 0.28 (0.09, 0.93) | 0.026 |
|  | ISS Stage 2 | 17/50 (34%) | 2.16 (0.95, 4.89) | 0.058 | 1.24 (0.61, 2.51) | 0.547 |
|  | ISS Stage 3 | 25/50 (50%) | 1.38 (0.62, 3.08) | 0.432 | 1.58 (0.81, 3.10) | 0.176 |
|  | Albumin < 3.5 g/dL | 28/50 (56%) | 5.63 (2.06, 15.42) | <.001 | 3.41 (1.60, 7.26) | <.001 |
|  | B2M >= 3.5 mg/L | 39/50 (78%) | 5.06 (1.18, 21.66) | 0.015 | 2.75 (1.06, 7.15) | 0.030 |
|  | B2M > 5.5 mg/L | 25/50 (50%) | 1.38 (0.62, 3.08) | 0.432 | 1.58 (0.81, 3.10) | 0.176 |
|  | Creatinine >= 1.5 mg/dL | 13/50 (26%) | 1.30 (0.55, 3.05) | 0.552 | 1.53 (0.75, 3.14) | 0.239 |
|  | CRP >= 8 mg/L | 18/50 (36%) | 1.60 (0.71, 3.63) | 0.256 | 1.98 (1.00, 3.92) | 0.046 |
|  | Hemoglobin < 10 g/dL | 33/50 (66%) | 2.94 (1.10, 7.89) | 0.025 | 2.26 (1.05, 4.85) | 0.032 |
|  | LDH >= 190 U/L | 19/50 (38%) | 1.15 (0.51, 2.60) | 0.734 | 1.21 (0.61, 2.39) | 0.578 |
|  | Platelet Count < 150 x 10^9/L | 22/50 (44%) | 1.53 (0.69, 3.39) | 0.288 | 1.51 (0.78, 2.94) | 0.221 |
|  | BMPC (Biopsy) >= 33% | 40/47 (85%) | 1.87 (0.55, 6.35) | 0.310 | 0.70 (0.30, 1.60) | 0.392 |
|  | BMPC (Aspirate) >= 33% | 36/47 (77%) | 1.98 (0.67, 5.89) | 0.211 | 0.88 (0.41, 1.89) | 0.739 |
|  | Baseline PET FL > 0 | 31/47 (66%) | 1.62 (0.67, 3.92) | 0.281 | 1.72 (0.82, 3.61) | 0.146 |
|  | Baseline PET FL > 3 | 24/47 (51%) | 1.80 (0.79, 4.09) | 0.154 | 1.63 (0.82, 3.22) | 0.156 |
|  | Baseline FL-SUV > 3.9 | 18/31 (58%) | 1.90 (0.66, 5.52) | 0.229 | 1.01 (0.45, 2.27) | 0.990 |
|  | Cytogenetic abnormalities | 37/50 (74%) | 1.81 (0.67, 4.88) | 0.238 | 0.95 (0.45, 1.97) | 0.882 |
|  | GEP CD-1 subgroup | 4/50 (8%) | 1.13 (0.26, 4.87) | 0.872 | 0.58 (0.14, 2.41) | 0.445 |
|  | GEP CD-2 subgroup | 1/50 (2%) | 0.00 (0.00, .) | 0.344 | 0.00 (0.00, .) | 0.241 |
|  | GEP HY subgroup | 3/50 (6%) | 0.86 (0.11, 6.45) | 0.884 | 0.37 (0.05, 2.71) | 0.308 |
|  | GEP LB subgroup | 2/50 (4%) | 0.76 (0.10, 5.66) | 0.785 | 1.92 (0.46, 8.06) | 0.366 |
|  | GEP MF subgroup | 7/50 (14%) | 0.76 (0.22, 2.58) | 0.660 | 0.98 (0.38, 2.55) | 0.967 |
|  | GEP MS subgroup | 14/50 (28%) | 0.92 (0.38, 2.23) | 0.849 | 0.97 (0.47, 2.03) | 0.943 |
|  | GEP PR subgroup | 19/50 (38%) | 1.48 (0.67, 3.26) | 0.331 | 1.56 (0.79, 3.07) | 0.196 |
| Multivariate | Albumin < 3.5 g/dL | 28/50 (56%) | 5.63 (2.06, 15.42) | <.001 | 3.41 (1.60, 7.26) | <.001 |
| HR- Hazard Ratio, 95% CI- 95% Confidence Interval, P-value from Score Chi-Square Test in Cox Regression NS2- Multivariate results not statistically significant at 0.05 level. All univariate p-values reported regardless of significance. Multivariate model uses stepwise selection with entry level 0.1 and variable remains if meets the 0.05 level. A multivariate p-value greater than 0.05 indicates variable forced into model with significant variables chosen using stepwise selection. | | | | | | |
